# SARS-CoV-2 Disinfection of Air and Surface Contamination by TiO_2_ Photocatalyst-Mediated Damage to Viral Morphology, RNA, and Protein

**DOI:** 10.3390/v13050942

**Published:** 2021-05-20

**Authors:** Ryosuke Matsuura, Chieh-Wen Lo, Satoshi Wada, Junichi Somei, Heihachiro Ochiai, Takeharu Murakami, Norihito Saito, Takayo Ogawa, Atsushi Shinjo, Yoshimi Benno, Masaru Nakagawa, Masami Takei, Yoko Aida

**Affiliations:** 1 Laboratory of Global Infectious Diseases Control Science, Graduate School of Agricultural and Life Sciences, The University of Tokyo, Tokyo 113-8657, Japan; oinari.atsuage@gmail.com (R.M.); rogerwen80@gmail.com (C.-W.L.); 2 Division of Hematology and Rheumatology, Department of Medicine, Nihon University School of Medicine, Tokyo 173-8610, Japan; swada@riken.jp (S.W.); somei.junichi@kaltec.co.jp (J.S.); ochiai.heihachiro@kaltec.co.jp (H.O.); nakagawa.masaru@nihon-u.ac.jp (M.N.); takei.masami@nihon-u.ac.jp (M.T.); 3 Laboratory of Global Animal Resource Science, Graduate School of Agricultural and Life Sciences, The University of Tokyo, Tokyo 113-8657, Japan; 4 Photonics Control Technology Team, RIKEN Center for Advanced Photonics, Saitama 351-0198, Japan; takeharu.murakami@riken.jp (T.M.); norihito@riken.jp (N.S.); pogawa@riken.jp (T.O.); kaminari@sfc.keio.ac.jp (A.S.); 5 Kaltech Co., Ltd., Osaka 541-0059, Japan; 6 Benno Laboratory, Baton Zone Program, RIKEN Cluster for Science, Technology and Innovation Hub, Saitama 351-0198, Japan; benno828@riken.jp

**Keywords:** SARS-CoV-2 inactivation, TiO_2_ photocatalyst, aerosol, RNA damage, viral morphology disruption, viral protein damage

## Abstract

SARS-CoV-2 is the causative agent of COVID-19, which is a global pandemic. SARS-CoV-2 is transmitted rapidly via contaminated surfaces and aerosols, emphasizing the importance of environmental disinfection to block the spread of virus. Ultraviolet C radiation and chemical compounds are effective for SARS-CoV-2 disinfection, but can only be applied in the absence of humans due to their toxicities. Therefore, development of disinfectants that can be applied in working spaces without evacuating people is needed. Here we showed that TiO_2_-mediated photocatalytic reaction inactivates SARS-CoV-2 in a time-dependent manner and decreases its infectivity by 99.9% after 20 min and 120 min of treatment in aerosol and liquid, respectively. The mechanistic effects of TiO_2_ photocatalyst on SARS-CoV-2 virion included decreased total observed virion count, increased virion size, and reduced particle surface spike structure, as determined by transmission electron microscopy. Damage to viral proteins and genome was further confirmed by western blotting and RT-qPCR, respectively. The multi-antiviral effects of TiO_2_-mediated photocatalytic reaction implies universal disinfection potential for different infectious agents. Notably, TiO_2_ has no adverse effects on human health, and therefore, TiO_2_-induced photocatalytic reaction is suitable for disinfection of SARS-CoV-2 and other emerging infectious disease-causing agents in human habitation.

## 1. Introduction

Severe acute respiratory syndrome coronavirus 2 (SARS-CoV-2) is a novel human coronavirus that causes coronavirus disease 2019 (COVID-19), which has had an unprecedented impact on modern human civilization [1] and resulted in more than 1.7 million deaths globally, as of late December 2020. SARS-CoV-2 belongs to a group of pathogenic enveloped viruses with positive single stranded RNA genomes encoding spike (S), envelope (E), membrane (M), and nucleocapsid (N) proteins, which are structural proteins required for producing structurally complete viral particles, and nonstructural proteins [2]. S protein mediates attachment of the virus to the host cell-surface receptor angiotensin-converting enzyme 2 (ACE2) and subsequent fusion between the viral and host cellular membranes to facilitate viral entry into the host cell [3]. N is the only protein that functions primarily to bind the RNA genome, constituting the nucleocapsid [3].

SARS-CoV-2 is believed to be transmitted through airborne route in addition to direct contact and droplet modes [4]. Thus, understanding the contaminations of aerosol and surfaces by SARS-CoV-2 is crucial to plan effective preventive measures and disrupt its transmission via environmental routes. For example, studies on the stability of SARS-CoV-2 have shown that it is viable in aerosols for at least 3 h [5]. Furthermore, SARS-CoV-2 is more stable on plastic and stainless steel than on copper and cardboard, and viable virus have been detected up to 72 h after application to these surfaces; the estimated median half-life of SARS-CoV-2 is approximately 5.6 h on stainless steel and 6.8 h on plastic [5]. However, the relationship of transmission with the distribution and pattern of environmental contamination by SARS-CoV-2 remains unclear.

Inactivating SARS-CoV-2 in the environment is crucial for controlling its transmission. Ethanol, isopropanol, povidone iodine, sodium hypochlorite, and alkyl dimethyl benzyl ammonium chloride inactivate SARS-CoV-2 in liquids [6,7,8,9] and on the skin as well as abiotic surfaces and tools [10]. In addition, SARS-CoV-2 is susceptible to physical treatments, including exposure to heat, acidity, and ultraviolet (UV) radiation. Exposure to UV light is a direct antimicrobial approach [11] with well-established efficacy against different strains of airborne viruses [12], and UVC effectively inactivates SARS-CoV-2 [13,14]. However, the use of UV lamps to disinfect public spaces occupied by humans is hazardous to their health because direct exposure to UV wavelengths can damage the skin and eyes [15]. Thus, the aforementioned methods are not suitable for use in living environments to eliminate SARS-CoV-2 from air.

The photocatalytic reaction of titanium dioxide (TiO_2_) is useful for disinfecting surfaces, air, and water. Indeed, the photocatalytic reaction of TiO_2_ kills microorganisms, such as bacteria and fungi, and inactivates influenza virus, hepatitis C virus, vesicular stomatitis virus, enterovirus, herpes virus, Zika virus, human coronavirus, bovine coronavirus, human norovirus, murine norovirus, SARS coronavirus, and bacteriophage [16,17,18,19,20,21,22,23]. Furthermore, it has previously been reported that phage B1 present in aerosols are effectively inactivated by the photocatalytic reaction of TiO_2_ [24]. Irradiation with visible or UV light activates the photocatalytic reaction of TiO_2_, generating reactive oxygen species (ROS), such as hydroxyl (·OH) and superoxide radicals (O_2_^−^), on the surface of TiO_2_ [16]. These radicals have strong oxidizing power and mineralize organic compounds. However, the wide bandgap (larger than 3 eV) of TiO₂ restricts its application to the UV region making it inefficient for energy conversion from visible light [25]. Narrowing the bandgap of TiO₂ through doping with other materials is important for improving the usage of TiO₂ under visible light [26]. For example, silicane (SiH) and TiO₂ composite, with a lower bandgap (2.082 eV), is an ideal material for the visible-light photoexcitation of electron-hole pairs [27]. These hybrid materials broaden the spectrum of radiations that can be used to initiate photocatalytic reaction and will enhance the applicability and cost-effectiveness of TiO₂-based disinfection to combat infectious diseases. Indeed, it has been recently reported that photocatalyzed TiO_2_ inactivates SARS-CoV-2 in liquid [18]. However, the underlying mechanisms are not clear.

Controlling the transmission of SARS-CoV-2 is an important preventive measure to curb the increase in COVID-19 cases. Although the transmission of SARS-CoV-2 via aerosols has been confirmed, photocatalytic disinfection of SARS-CoV-2 in aerosols using TiO_2_ has not yet been evaluated. In this study, we investigated the inactivation of SARS-CoV-2 in liquid using light emitting diode (LED)-activated TiO_2_ immobilized on a glass sheet. Furthermore, we demonstrated the inactivation of SARS-CoV-2 sprayed with nebulizer as aerosols into a 120 L acrylic box by photocatalytic reaction using an air cleaner with TiO_2_-coated sheet and LED light. Finally, we clarified the disinfecting mechanism of TiO_2_ by observing virion morphology using electron microscopy, and performing reverse transcription quantitative PCR and immunoblot to detect damaged viral RNA and proteins.

## 2. Materials and Methods

### 2.1. Cells and Viruses

Vero E6/TMPRSS2 cells (Japanese Collection of Research Bioresources no. JCRB1819) were cultured in Dulbecco’s Modified Eagle’s Medium (DMEM, Thermo Fisher Scientific, Waltham, MA, USA) supplemented with 10% heat-inactivated fetal bovine serum (FBS, Sigma-Aldrich, St. Louis, MO, USA), 1% penicillin/streptomycin/glutamine (PSG), and 2% G418 (Thermo Fisher Scientific) at 37 °C with 5% CO_2_. SARS-CoV-2 (SARS-CoV-2/JPN/TY/WK-521 strain) was kindly gifted by the National Institute of Infectious Diseases of Japan [28]. SARS-CoV-2 was propagated using Vero E6/TMPRSS2 cells cultured in DMEM containing 2% FBS and 1% PSG, and titrated using 50% tissue culture infective dose (TCID_50_) assays as follows: Vero E6/TMPRSS2 cells cultured in a 96-well plate (2 × 10^4^ cells per well) were infected with 100 μL of 10-fold serially diluted virus-containing infection medium (each dilution had 8 replicates) and incubated at 37 °C for 3 days. Following incubation, viral infection in each well was determined based on the virus-induced cell cytopathic effect. Detection limit of TCID_50_ assays was 1.0 TCID_50_/mL.

### 2.2. Inactivation of SARS-CoV-2 in Liquid by the LED-TiO_2_ Photocatalytic Reaction

Inactivation of the virus by photocatalyst was performed as described by Park et al. [29]. Glass fiber sheet was soaked in TiO_2_ dispersion and its surface was coated with TiO_2_ that was fixed by heat treatment. TiO_2_-coated sheet (3 cm × 3 cm) was placed in a dish with a diameter of 10 cm and LED light (405 nm) source was placed 6 cm above the dish. TiO_2_ photocatalyst was excited by 405 nm LED, which excites the photocatalyst at the same level as UV light. To confirm the effect of LED-TiO_2_ on SARS-CoV-2 infectivity, 1 mL of 1.0 × 10^5^ TCID_50_/mL SARS-CoV-2 titer was placed on the TiO_2_-coated sheet in the 10-cm dish. TiO_2_-coated sheet was exposed to LED light for 0, 30, 60, or 120 min to activate the photocatalytic reaction. After irradiation with LED light, SARS-CoV-2 was collected by adding 9 mL phosphate-buffered saline (PBS) to confirm the effect of TiO_2_. For transmission electron microscopy (TEM) and western blotting, 1 mL of 1.78 × 10^6^ TCID_50_/mL SARS-CoV-2 titer was placed on the TiO_2_-coated sheet and exposed to LED light. SARS-CoV-2 was then collected by adding 1 mL of Minimum Essential Media (Thermo Fisher Scientific) containing 2% FBS. To observe the effect of LED light on SARS-CoV-2 infectivity, the virus particles were directly placed on the 10-cm dish and exposed to LED light (405 nm) for 0, 30, 60, or 120 min. In addition, a control was prepared by directly placing SARS-CoV-2 on the 10-cm dish, which was then covered with aluminum foil to obstruct light. The LED-TiO_2_ photocatalyst-treated virus was then titrated using TCID_50_ assay.

### 2.3. TEM

On the TiO_2_-coated sheet, 1 mL of SARS-CoV-2 titer with 1.78 × 10^6^ TCID_50_/mL was placed. The LED-TiO_2_ photocatalytic reaction was activated by exposure to LED light (405 nm) for 120 min. SARS-CoV-2 was directly placed on 10-cm dishes and was exposed to LED light in Light group and left unexposed in control group. After incubation, 100 μL of virus sample was mixed with 100 μL of 2.5% glutaraldehyde for TEM negative staining. For TEM sample preparation, a droplet of virus sample was loaded on a carbon-film grid and incubated for 10 s. Next, the grid was partially dried, and a droplet of 2% uranyl acetate staining solution was added, followed by incubation for 10 s. Finally, the excess liquid was removed with filter paper and the grid was dried at room temperature before obtaining images using HITACHI H-7600 electron microscope (Hitachi Global Life Solutions, Inc., Tokyo, Japan) at 100 kV.

### 2.4. Western Blotting

On the TiO_2_-coated sheet, 1 mL SARS-CoV-2 titer (1.78 × 10^6^ TCID_50_/mL) was placed and LED-TiO_2_ photocatalytic reaction was activated by exposure to LED light (405 nm) for 0, 30, 60, or 120 min. After photocatalytic reaction, 20 µL of virus-containing medium was mixed with 5 µL sample buffer (0.15 M Tris-HCl, 10% sodium dodecyl sulfate (SDS), 30% glycerol, and 0.5% bromophenol blue) and heated at 100 °C for 5 min. Then, 15 µL denatured virus solution was loaded on 8% (for S protein detection) or 10% (for N protein detection) SDS-polyacrylamide gel and electrophoresed with a running buffer containing 0.3% Tris, 0.1% SDS, and 1.44% glycine. The proteins were then transferred onto a polyvinylidene difluoride membrane (Millipore, Billerica, MA, USA) using a Trans-Blot Turbo apparatus (Bio-Rad, Hercules, CA, USA). The membrane was blocked with 5% non-fat skim milk and then incubated overnight with anti-SARS-CoV-2 Spike monoclonal antibody (Mab) (1A9) (1:1000; GENETEX, Irvine, CA, USA) and anti-SARS-CoV-2 Nucleocapsid Mab (6H3) (1:2000; GENETEX) at 4 °C. After washing with PBS containing 1% TWEEN 20, the membranes were incubated with horseradish peroxidase-conjugated AffiniPure goat anti-mouse immunoglobulin G (IgG) (1:2000; Jackson ImmunoResearch, West Grove, PA, USA) at room temperature for 1 h. Signals were visualized after treating the membrane with SuperSignal™ West Pico PLUS Chemiluminescent Substrate (Thermo Fisher Scientific). Images were acquired using a WSE-6100 LuminoGraph I (ATTO CORPORATION, Tokyo, Japan). Densities of bands were analyzed using CSAnlyzer4 software (ATTO CORPORATION).

### 2.5. Reverse Transcription Quantitative PCR (RT-qPCR)

The viral RNA of SARS-CoV-2 was extracted using QIAamp Viral RNA Mini Kit (Qiagen, Hilden, Germany) according to the manufacturer’s instructions. For RT-qPCR, 5 μL RNA was first reverse transcribed with SARS-CoV-2 4R primer (CTCTTCCATATAGGCAGCTCT) using SuperScript™ III First-Strand Synthesis System (Invitrogen, Foster, CA, USA) according to the manufacturer’s instructions. In total, 1.25 μL cDNA was used for qPCR analysis with the QuantiTect Probe RT-PCR Kit (Qiagen). The reaction mixture contained 600 nM forward primer (CACATTGGCACCCGCAATC), 800 nM reverse primer (GAGGAACGAGAAGAGGCTTG), and 200 nM TaqMan probe (FAM-ACTTCCTCAAGGAACAACATTGCCA-QSY); the reaction was conducted in an Applied Biosystems 7500 Fast Real-Time PCR system (Thermo Fisher Scientific) with the following thermal cycling conditions: 50 °C for 30 min; 95 °C for 15 min; and 45 cycles of 95 °C for 15 s and 60 °C for 1 min, as described previously [30]. Samples were evaluated in triplicate and data analysis was performed using the comparative CT method (2^−∆∆CT^).

### 2.6. Inactivation of SARS-CoV-2 in Aerosol by LED-TiO_2_ Photocatalytic Reaction

Using a nebulizer (Omron Co., Ltd., Kyoto, Japan), 2.3 mL of 1.78 × 10^6^ TCID_50_/mL SARS-CoV-2 titer was sprayed as aerosols into a 120 L acrylic box (50 cm × 40 cm × 60 cm) for 10 min. Diameter of droplet from nebulizer was measured using a particle counter (P8-506-30; Airy Technology, Stoughton, MA, USA). The air containing SARS-CoV-2 as aerosols was circulated in the acrylic box for 0, 5, 10, 15, or 20 min using an air cleaner (KL-W01; Kaltech Co., LTD., Osaka, Japan), either with both TiO_2_-coated sheet and LED light or with LED light alone. KL-W01 contains a TiO_2_-coated sheet (25 cm × 25 cm) and 48 LED light (405 nm) source. LED light was placed 2 cm above the TiO_2_-coated sheet and the TiO_2_-coated sheet was irradiated with 10 mW of light. As control, SARS-CoV-2 in aerosol was left without circulation using an air cleaner for 0, 5, 10, 15, or 20 min. SARS-CoV-2 in aerosol was captured in gelatin membrane filters (Sartorius, Gottingen, Germany) using MD8 microbiological sampler (Sartorius) with 120 L air; the gelatin membrane filter was subsequently molten in MEM containing 2% FBS. LED-TiO_2_ photocatalyst-treated virus was then titrated by TCID_50_ assay.

### 2.7. Statistical Analysis

Two-way analysis of variance (ANOVA) with Dunnett’s test was used to compare all samples with the sample obtained at 0 min for statistical determination. For TEM data analysis, ANOVA followed by Tukey’s test was performed to compare each group. *p* values < 0.05 were considered statistically significant. Linear regression analysis was performed to determine (1) the relation between viral infectivity and LED-TiO_2_ photocatalytic reaction duration; (2) the relation between viral infectivity and the level of RNA; and (3) the relation between viral infectivity and the band intensities of viral proteins. All calculations were performed using R software (version 3.6.3, R Foundation for Statistical Computing, Vienna, Austria).

## 3. Results

### 3.1. LED-TiO_2_ Photocatalytic Inactivation of SARS-CoV-2 in Liquid

**Figure 1:**
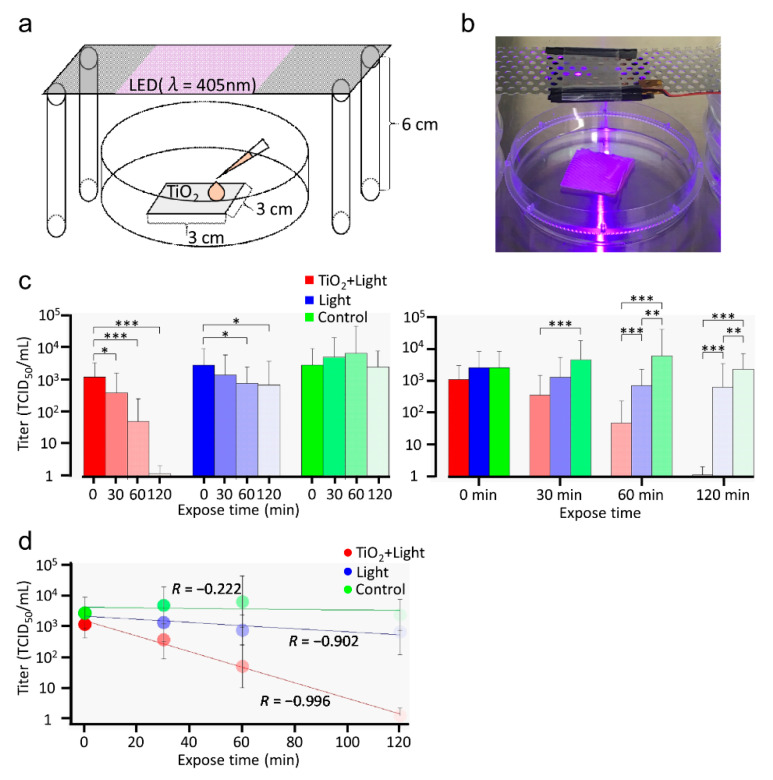
Inactivation of SARS-CoV-2 in liquid by LED-TiO_2_ photocatalytic reaction. Schematic diagram (**a**) and images (**b**) of TiO_2_-coated sheet (3 cm × 3 cm) placed a 10-cm dish and exposed to light emitting diode (LED) light with a wavelength of 405 nm placed 6 cm above the dish. In the TiO_2_ + Light group, SARS-CoV-2 (1 mL) titer (1.0 × 10^5^ TCID_50_/mL) was placed on the TiO_2_-coated sheet. In the Light and control groups, SARS-CoV-2 was directly placed on 10-cm dishes. In the TiO_2_ + Light and Light groups, SARS-CoV-2 were exposed to LED light for 0, 30, 60, or 120 min. Then, SARS-CoV-2 were collected by adding 9 mL PBS. (**c**) After the photocatalytic reaction, viral titer was confirmed by TCID_50_ assay. Each column and error bar represents the mean ± SD of the results for two independent experiments. All values in each group were compared with those of the 0 min sample by two-way ANOVA with Dunnett’s test (left panel). All values at each time point were analyzed by two-way ANOVA followed by Tukey’s test (right panel). Asterisk indicates a statistically significant difference (* *p* < 0.05; ** *p* < 0.01; *** *p* < 0.001). (**d**) Linear regression analysis was used to examine the correlation between LED-TiO_2_ photocatalytic reaction duration and SARS-CoV-2 infectivity. R indicates the Pearson correlation coefficient.

To determine whether the photocatalytic reaction of TiO_2_ can be used to disinfect surfaces contaminated with SARS-CoV-2, we first investigated the inactivation of SARS-CoV-2 in liquid using LED light (405 nm), both with and without TiO_2_. In the TiO_2_ + Light group, a 1.0 × 10^5^ TCID_50_/mL titer of SARS-CoV-2 was placed on a TiO_2_-coated sheet and then exposed to LED light for 0, 30, 60, or 120 min, as shown in Figure 1a,b. Our photocatalytic reaction was saturated by 1 mW of light. Therefore, light was exposed from a distance of 6 cm and TiO_2_-coated sheet was irradiated with approximately 1 mW of light. In the Light group, the viruses were directly placed on a 10-cm dish and irradiated with LED light. Moreover, viruses were incubated on a 10-cm dish without light as control. After LED-TiO_2_ photocatalytic reaction, the viral titer was monitored in Vero E6/TMPRSS2 cells using TCID_50_ assays. Interestingly, the infectivity of SARS-CoV-2 was significantly (*p* < 0.05 and *p* < 0.001) reduced after 30, 60, and 120 min of the photocatalytic reaction by LED-TiO_2_. Moreover, SARS-CoV-2 was inactivated almost to the detection limit after 120 min of this photo-catalytic reaction (Figure 1c). On the contrary, although the SARS-CoV-2 titer in the Light group was significantly (*p* < 0.05) reduced after 60 and 120 min of exposure to LED light, this decrease was not sufficient to completely inactivate SARS-CoV-2 (Figure 1c left panel). Notably, LED-TiO_2_ photocatalytic reaction was significantly (*p* < 0.001) more effective in inactivating SARS-CoV-2 than LED light alone after 60 and 120 min of reaction duration (Figure 1c right panel)_._ In the control group, the SARS-CoV-2 titer was not decreased. As shown in Figure 1d, the decreased infectivity of SARS-CoV-2 due to LED-TiO_2_ photocatalytic reaction showed exponential correlation with the reaction duration, indicating that the LED-TiO_2_ photocatalytic reaction inactivated SARS-CoV-2 in a time-dependent manner. Our results strongly suggested that LED-TiO_2_ photocatalytic reaction can efficiently inactivate SARS-CoV-2 in liquid.

### 3.2. Mechanism of LED-TiO_2_ Photocatalytic Reaction-Induced SARS-CoV-2 Inactivation

**Figure 2:**
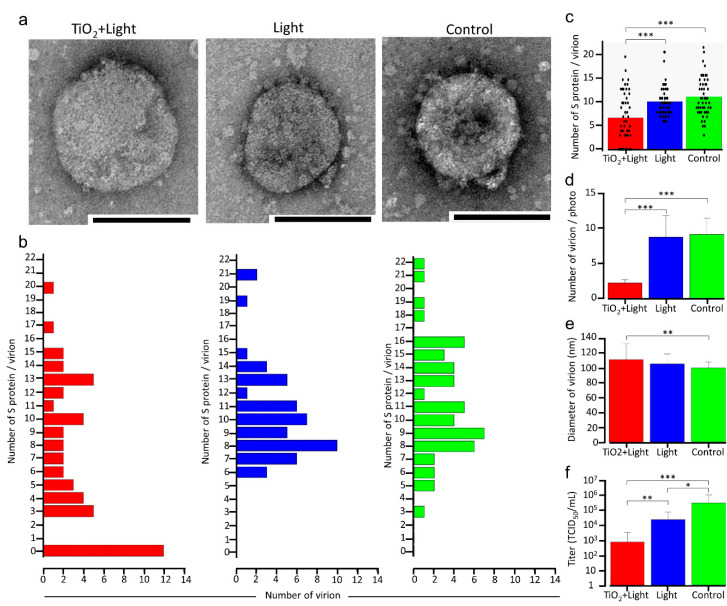
Changes in SARS-CoV-2 virion morphology due to LED-TiO_2_ photocatalytic reaction. (**a**) SARS-CoV-2 (1 mL) titer of 1.78 × 10^6^ TCID_50_/mL was placed on TiO_2_-coated sheet and subjected to photocatalytic reaction for 120 min before TEM imaging. Representative virion images in the TiO_2_ + Light, Light, and control groups are shown. Bar = 100 nm. (**b**) Number of S proteins on single virions in individual TEM images of the TiO_2_ + Light, Light, and control groups was counted, and distribution and mean number of S protein/virion are shown. *n* = 50/group. (**c**) Each dot represents a value of S protein of each virion in (**b**). (**d**) Virion number in an area of 170 μm^2^ in an individual TEM image is shown, *n* = 10. (**e**) Diameter of single virion in an individual TEM image is shown. *n* = 40. (**f**) Viral titer in each group was confirmed by TCID_50_ assay. Each column and error bar represents the mean ± SD of results. All values were analyzed by two-way ANOVA followed by Tukey’s test. Asterisk indicates a statistically significant difference (* *p* < 0.05; ** *p* < 0.01; *** *p* < 0.001).

It has been previously reported that photocatalytic reactions decompose the bacterial cell membrane [31,32,33]. Therefore, to clarify the mechanism of SARS-CoV-2 inactivation by a photocatalytic reaction, the initial experimental design aimed at observing the virion morphology of SARS-CoV-2 using TEM. A high viral titer (1.78 × 10^6^ TCID_50_/mL) of SARS-CoV-2 was irradiated with LED light, with or without the TiO_2_-coated sheet, for 120 min. As shown in Figure 2f, SARS-CoV-2 infectivity decreased significantly by 2.625 and 1.125 log_10_ in the TiO_2_ + Light and Light groups, respectively, compared with that in the control group; however, some replication activity remained in all groups. Surprisingly, 24% of the SARS-CoV-2 virion particles lost their surface S protein after LED-TiO_2_ photocatalytic reaction (Figure 2a,b); it was assumed that the use of a high titer of the virus for TEM led to incomplete SARS-CoV-2 inactivation by the photocatalytic reaction. In contrast, all virion particles in the Light and control groups expressed the S protein and no significant differences in virion morphology were detected. These results showed that the LED-TiO_2_ photocatalytic reaction could significantly reduce the number of S protein expressed on the virion surface (Figure 2c). Furthermore, the number of viral particles in a 170 μm^2^ area of the individual TEM images (*n* = 10) was significantly decreased in the TiO_2_ + Light group, compared with those in the Light and control groups (Figure 2d), indicating that not only S protein but also the virion particles were damaged. Moreover, the TEM images (*n* = 40) revealed that the diameters of viral particles increased significantly after the LED-TiO_2_ photocatalytic reaction, when compared with those in the control group (Figure 2e), indicating that the damage to the virion membrane may have caused the change in SARS-CoV-2 size. Thus, our results suggested that LED-TiO_2_ photocatalytic reaction changed virion morphology.

**Figure 3:**
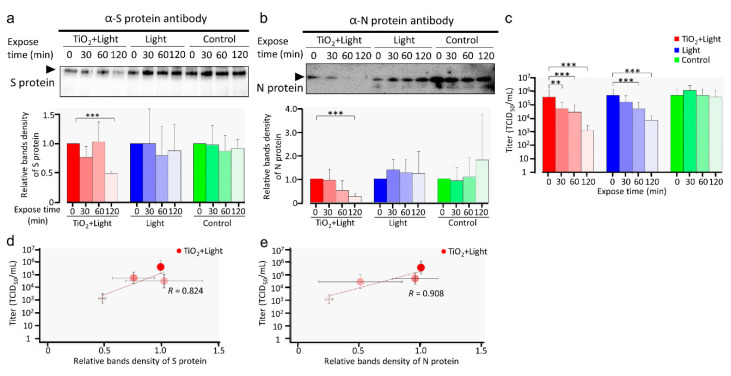
Damage to SARS-CoV-2 viral proteins by LED-TiO_2_ photocatalytic reaction. (**a**–**c**) SARS-CoV-2 (1 mL) titer of 1.78 × 10^6^ TCID_50_/mL was placed on TiO_2_-coated sheet and subjected to photocatalytic reaction for 0, 30, 60, and 120 min before western blotting for S (**a,** upper panel) and N proteins (**b**, upper panel) of SARS-CoV-2. Original blots can be seen in Figure S1. Positions of S and N proteins are indicated. Intensities of bands were analyzed using CSAnlyzer4 software and the quantitative results are shown (**a**,**b**, lower panels). Data in the plot represent the mean ± standard error (SD) of three or four replicates. Each viral titer sample was examined by TCID_50_ assay (**c**). (**d**,**e**) Linear regression analysis between relative SARS-CoV-2 infectivity and band intensity of S (**d**) and N (**e**) proteins. Each column and error bar represent the mean ± SD of results for two experiments. R indicates the Pearson correlation coefficient. All values were analyzed by two-way ANOVA with Dunnett’s test. Asterisk indicates a statistically significant difference (** *p* < 0.01; *** *p* < 0.001).

TEM analysis showed that the S protein was degraded by the photocatalytic reaction. Next, after 0 to 120 min of photocatalytic reaction, we detected S and N proteins by western blotting; 180 kDa and 55 kDa bands represented S and N proteins, respectively (Figure 3a,b upper panel). The band intensities of both S and N proteins in the TiO_2_ + Light group decreased in a time-dependent manner (Figure 3a,b). In contrast, the band intensities of these proteins in the Light and control groups showed no significant differences. These results suggested that the S and N proteins of SARS-CoV-2 were degraded by TiO_2_-induced photocatalytic reaction, corroborating the results of TEM. The titers (Figure 3c) of the virus samples were determined. Linear regression analysis strongly indicated a correlation between viral infectivity and the relative blot intensities of S and N proteins (*R* = 0.824 and 0.908, respectively) after LED-TiO_2_ photocatalytic reaction (Figure 3d,e), suggesting that the degradation of both viral proteins is critical for SARS-CoV-2 inactivation.

**Figure 4:**
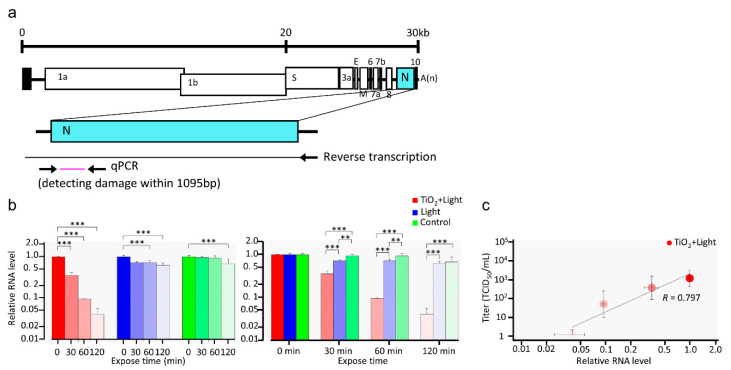
Damage to SARS-CoV-2 RNA by LED-TiO_2_ photocatalytic reaction. (**a**) Schematic diagram of RT-qPCR primer-binding sites. (**b**) SARS-CoV-2 (1 mL) titer of 1.0 × 10^5^ TCID_50_/mL was placed on the TiO_2_-coated sheet and subjected to photocatalytic reaction for 0, 30, 60, and 120 min, and then viral RNA was extracted and measured by RT-qPCR. The relative RNA level compared to that in samples obtained at 0 min after LED light irradiation was calculated. All values in each group were compared with the 0 min sample by two-way ANOVA with Dunnett’s test (left panel). All values at each time point were analyzed by two-way ANOVA followed by Tukey’s test (right panel). (**c**) Linear regression analysis between SARS-CoV-2 infectivity (Figure 1) and RNA level. Each column and error bar represent the mean ± SD of results for two experiments. Asterisk indicates a statistically significant difference (** *p* < 0.01; *** *p* < 0.001). R indicates the Pearson correlation coefficient.

Previous studies on bacteriophage and human norovirus have shown that TiO_2_ not only damages viral protein but also the viral genome [29,34]. Therefore, to investigate whether LED-TiO_2_ photocatalytic reaction induces damage to SARS-CoV-2 RNA, RT-qPCR was performed to detect viral RNA after different photocatalytic reaction durations ranging from 0 to 120 min. As shown in Figure 4a, viral RNA was reverse transcribed from the 3′-terminal to the 5′-terminal of *N* gene, covering 1095 bp. As shown in Figure 4b, the level of viral RNA was significantly decreased after LED-TiO_2_ photocatalytic reaction in a time-dependent manner; after 120 min of the photocatalytic reaction, SARS-CoV-2 RNA level was decreased by 96.0%. Notably, the level of SARS-CoV-2 RNA was correlated with the viral titer derived from Figure 1c (Figure 4c). In addition, exposure to LED-light for 120 min without TiO_2_ photocatalytic reaction reduced SARS-CoV-2 RNA level by only 37.0%, indicating that the decrease of SARS-CoV-2 RNA by LED light alone is lower than that by LED-TiO_2_ photocatalytic reaction. This might be the reason why LED without TiO_2_ only slightly decreased viral infectivity. Thus, our results suggested that LED-TiO_2_ photocatalytic reaction inactivated SARS-CoV-2 by damaging its genome as well as proteins and virion membrane in liquids.

### 3.3. LED-TiO_2_ Photocatalytic Reaction Inactivated SARS-CoV-2 in Aerosols

**Figure 5:**
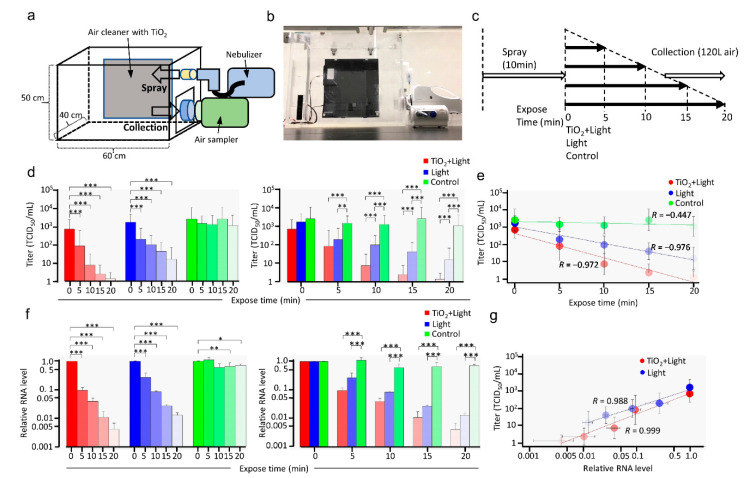
Inactivation of SARS-CoV-2 in aerosols by LED-TiO_2_ photocatalytic reaction. Schematic diagram (**a**), image (**b**), and time course (**c**) of the inactivation of SARS-CoV-2 in the aerosol test system. SARS-CoV-2 (2.3 mL) titer of 1.78 × 10^6^ TCID_50_/mL was sprayed as aerosol into a 120 L acrylic box using nebulizer for 10 min. Then, air cleaner with TiO_2_-coated sheet and LED light or only LED light was used to circulate SARS-CoV-2 in aerosols. As control, SARS-CoV-2 in aerosol were left without circulation using air cleaner. SARS-CoV-2 in aerosols were captured in gelatin filter using MS8 microbiological sampler with 120 L and the gelatin membrane filter was molten in MEM containing 2% FBS. (**d**) Viral titer of SARS-CoV-2 collected from the gelatin membrane filter was assessed by TCID_50_ assay. All values in each group were compared with those of the sample obtained at 0 min by two-way ANOVA with Dunnett’s test (left panel). All values at each time point were analyzed by two-way ANOVA followed by Tukey’s test (right panel). Asterisk indicates a statistically significant difference (* *p* < 0.05; ** *p* < 0.01; *** *p* < 0.001). (**e**) Linear regression analysis to examine the correlation between LED-TiO_2_ photocatalytic reaction duration and SARS-CoV-2 infectivity. R indicates the Pearson correlation coefficient. (**f**) Viral RNA was extracted from the gelatin membrane filter and detected by RT-qPCR. All values in each group were compared with those of the 0 min sample by two-way ANOVA with Dunnett’s test (left panel). All values at each time point were analyzed by two-way ANOVA followed by Tukey’s test (right panel). Asterisk indicates a statistically significant difference (* *p* < 0.05; ** *p* < 0.01; *** *p* < 0.001). (**g**) Linear regression analysis to examine the correlation between SARS-CoV-2 infectivity and relative viral RNA level. Each column and error bar represent the mean ± SD of results for two experiments. R indicates the Pearson correlation coefficient.

Preventing aerosol-mediated transmission of SARS-CoV-2 is essential to control the COVID-19 pandemic. However, it is still unclear whether LED-TiO_2_ photocatalytic reaction could inactivate SARS-CoV-2 in aerosols. To address this question, SARS-CoV-2 was sprayed as aerosols into a 120 L acrylic box with a nebulizer and circulated through a TiO_2_-coated sheet using an air cleaner and exposed to LED light (405 nm) for 0 to 20 min (Figure 5a–c). After circulation, the SARS-CoV-2 in aerosols were captured in a gelatin membrane filter and subjected to TCID_50_ assay. Diameter of droplet without SARS-CoV-2 from nebulizer, measured using a particle counter, were found to be 2.5 and 5.0 μm, which is close to the particle size generated by coughing [35]. This result indicated that aerosol sprayed by the nebulizer can imitate those generated by natural cough.

As shown in Figure 5d, SARS-CoV-2 infectivity was significantly inactivated in a time-dependent manner by LED-TiO_2_ photocatalytic reaction for 5, 10, 15, and 20 min, suggesting that the replication of almost all virions in aerosols ceased after LED-TiO_2_ photocatalytic reaction for 20 min. In contrast, although the circulating SARS-CoV-2 titer decreased in a time-dependent manner after exposure to LED light alone, without TiO_2_, not all viruses were inactivated. Notably, the SARS-CoV-2 titer was not decreased in the control group. The level of LED-TiO_2_ photocatalytic reaction-induced SARS-CoV-2 inactivation was significantly (*p* < 0.001) higher than that of LED light alone after 20 min (Figure 5d)_._ Linear regression analysis further indicated that the decrease in viral titer after LED-TiO_2_ photocatalytic reaction was exponentially correlated with reaction time and that LED irradiation for 18.75 min fully inhibited SARS-CoV-2 infectivity in aerosols (*R* = 0.972) (Figure 5e).

Next, the damage to viral genome was assessed by RT-qPCR covering 1095 bp of the *N* gene region (Figure 5f). Both LED-TiO_2_ photocatalytic reaction and LED light alone significantly (*p* < 0.001) decreased the viral RNA levels in a time-dependent manner (Figure 5f left panel), with no significant differences due to the different exposure durations (Figure 5f right panel). Furthermore, linear regression analysis showed that the viral RNA level was strongly correlated with SARS-CoV-2 titer (Figure 5d,g), indicating that damage to the viral genome was a mechanism of SARS-CoV-2 inactivation by LED-TiO_2_ photocatalytic reaction and LED light alone. Taken together, the results of this study showed that LED-TiO_2_ photocatalytic reaction inactivated SARS-CoV-2 in aerosols.

## 4. Discussions

Here we confirmed that the LED-TiO_2_ photocatalytic reaction effectively inactivated SARS-CoV-2. Indeed, the present study is the first to report that TiO_2_ photocatalytic reaction for 20 min inactivated 99.9% SARS-CoV-2 in aerosols. Furthermore, our results provide evidence that TiO_2_ photocatalytic reaction for 120 min inactivates 99.9% SARS-CoV-2 in liquid, consistent with recent reports showing that TiO_2_ photocatalytic reaction kills microorganisms, such as bacteria and fungi, and inactivates SARS-CoV-2, influenza virus, norovirus, SARS coronavirus, and bacteriophage [16,17,18,21,22,29,34]. Thus, it is possible that TiO_2_ effectively inactivates many kinds of viruses. Moreover, we found that the LED-TiO_2_ photocatalytic reaction inactivated SARS-CoV-2 via viral protein degradation, virion membrane damage, and RNA damage. Naked viruses were observed in TEM images and S protein degradation was detected by western blotting. In addition, the levels of N protein, which packages and protects the viral genome by binding with it, and the surface S protein on viral particles were decreased by the LED-TiO_2_ photocatalytic reaction. Interestingly, TEM showed that the viral diameter was enlarged and the number of virus particles was decreased after the photocatalytic reaction. These results suggested that TiO_2_ damaged the virion membrane, thereby destroying the virions and sequentially damaging viral RNA and proteins. Thus, TiO_2_-induced photocatalytic reaction affected entire SARS-CoV-2 particles and inactivated the virus.

Influenza virus surface proteins, HA and NA, are degraded by photocatalytic reactions, thereby inactivating the virus [17]. The capsid protein of *Lactobacillus* phage PL1 is also damaged by ROS produced via photocatalytic reactions, and the phage DNA inside the viral particles is subsequently damaged [34]. Furthermore, the bacteriophage protein MS2 and norovirus protein GI.1 are oxidized via photocatalytic reactions [29]. The present study demonstrated that LED-TiO_2_ photocatalytic reaction caused degradation of S protein that is essential for binding to viral receptor ACE2, thereby inhibiting viral penetration into the cells as well as caused degradation of N protein that is essential for viral life cycle, thereby decreasing the number of viral replicates; the levels of both proteins were correlated with infectivity, indicating that viral protein degradation is the main mechanism of SARS-CoV-2 inactivation in this approach. Moreover, TEM showed that SARS-CoV-2 lost the S protein on their surface and became naked particles after the photocatalytic reaction, which generates ROS on the surface TiO_2_ [16]. Thus, the S protein on the surface of viral particles is more susceptible to photocatalytic reaction than the nucleocapsid protein located inside the viral particle; this may underlie the production of naked viral particles by LED-TiO_2_ photocatalytic reaction.

It has been reported that TiO_2_ peroxidizes lipid bilayer of cells [31]. Photocatalytic reaction ruptures the lipid bilayer in *Pseudomonas aeruginosa* and alters the morphology of *Escherichia coli* [32,33]. In cancer cells, TiO_2_ photocatalytic reaction oxidizes the cell membrane, increasing permeability to Ca^2+^ [36]. Moreover, cell membrane oxidation increases permeability to water [37,38]. These reports suggest that photocatalytic reactions damage viral membranes. Our results showed that SARS-CoV-2 virions were significantly enlarged by LED-TiO_2_ photocatalytic reaction, likely because the oxidation of their lipid bilayer membranes increases permeability to water and ions, similar to that observed in cell membrane damage [36,37,38]. It follows that the number of viral particles was significantly decreased. In addition, the morphology of the viral membrane of SARS-CoV-2 significantly changed as well, similar to that observed in *Escherichia coli* [32,33], by LED-TiO_2_ photocatalytic reaction. These results suggested that the SARS-CoV-2 membrane was damaged by LED-TiO_2_ photocatalytic reaction.

Photocatalytic reactions have been reported to reduce viral RNA levels of phage MS2 and human norovirus [29]. In this study, LED-TiO_2_ photocatalytic reaction inactivated SARS-CoV-2 via damage to its RNA in both liquid and aerosols. It has recently been reported that light, such as UVC light, damages SARS-CoV-2 RNA, thereby inactivating the virus [13]. In this study, LED light (405 nm) alone reduced SARS-CoV-2 RNA levels in both liquid and aerosols, subsequently impairing viral infectivity. However, the reduction of RNA levels by LED light alone was lesser than that by LED-TiO_2_ photocatalytic reaction in this study, suggesting that the reaction caused RNA damage.

A study reported that 1 mg/mL of bovine serum albumin (BSA) inhibits the photocatalytic inactivation of influenza virus in liquid and reduces the efficacy by one hundredth, whereas 0.1 mg/mL of BSA did not affect the efficacy of inactivation of influenza virus in liquid [17]. In our study, SARS-CoV-2 was prepared with approximately 1 mg/mL FBS (2%). To avoid the influence of FBS, SARS-CoV-2 was diluted in PBS containing 0.05 mg/mL of FBS, and this was used for evaluating SARS-CoV-2 inactivation in liquid. Notably, although SARS-CoV-2 containing 1 mg/mL of FBS was used in the aerosol experiment, all virions in aerosols ceased after LED-TiO_2_ photocatalytic reaction for 20 min. Therefore, it is considered that the effect of FBS on the inactivation of SARS-CoV-2 in aerosol was limited.

Recent studies have reported that the D614G mutation in S protein of SARS-CoV-2 increases infectivity [39,40], and suggested that this mutation of S protein, which binds to ACE2 and initiates viral entry into host cells, is closely related with SARS-CoV-2 infectivity. Our results further showed that the photocatalytic reaction by TiO_2_ degraded S protein, which is important for infection, suggesting that photocatalytic reaction can inactivate SARS-CoV-2 regardless of mutation in the S protein.

The present study showed the potential of LED-TiO_2_ photocatalytic reaction to inactivate SARS-CoV-2. Unlike other light-based inactivation methods, such as UVC that inactivates viruses but is harmful to the human body, TiO_2_-photocatalytic reaction efficiently inactivates SARS-CoV-2 without toxicity to humans. Therefore, TiO_2_-photocatalytic reaction is appropriate for application in indoor environments to reduce the risk of SARS-CoV-2 transmission via aerosols. Notably, TiO_2_-induced photocatalytic reaction destroyed viral structure, proteins, and genome, suggesting universal inhibition of different types of infectious disease-causing agents by the multi-antiviral effects of TiO_2_-mediated photocatalytic reaction. Taken together, the present study suggests that TiO_2_-mediated photocatalytic reaction can be utilized to control SARS-CoV-2 transmission and mitigate the ongoing COVID-19 pandemic; moreover, it can potentially be applied against other newly emerging infectious agents.

## Supplementary Materials

The following are available online at https://www.mdpi.com/article/10.3390/v13050942/s1, Figure S1: Original image for blots.
