# Supplementary material for: SARS-CoV-2 Disinfection of Air and Surface Contamination by TiO2 Photocatalyst-Mediated Damage to Viral Morphology, RNA, and Protein
Source: Viruses. 2021 May 20;13(5):942. doi: 10.3390/v13050942 (PMC8161138; doi:10.3390/v13050942)
Supplement: Supplementary file 1 [file viruses-13-00942-s001.zip › viruses-1176387-supplementary.pdf]

Supplementary Materials

Title

SARS-CoV-2 disinfection by TiO2 photocatalyst via damage to viral morphology, RNA and protein

Author

Ryosuke Matsuura, Chieh-Wen Lo, Satoshi Wada, Junichi Somei, Heihachiro Ochiai, Takeharu Murakami, Norihito Saito, Takayo Ogawa, Atsushi Shinjo, Yoshimi Benno, Masaru Nakagawa, Masami Takei and Yoko Aida

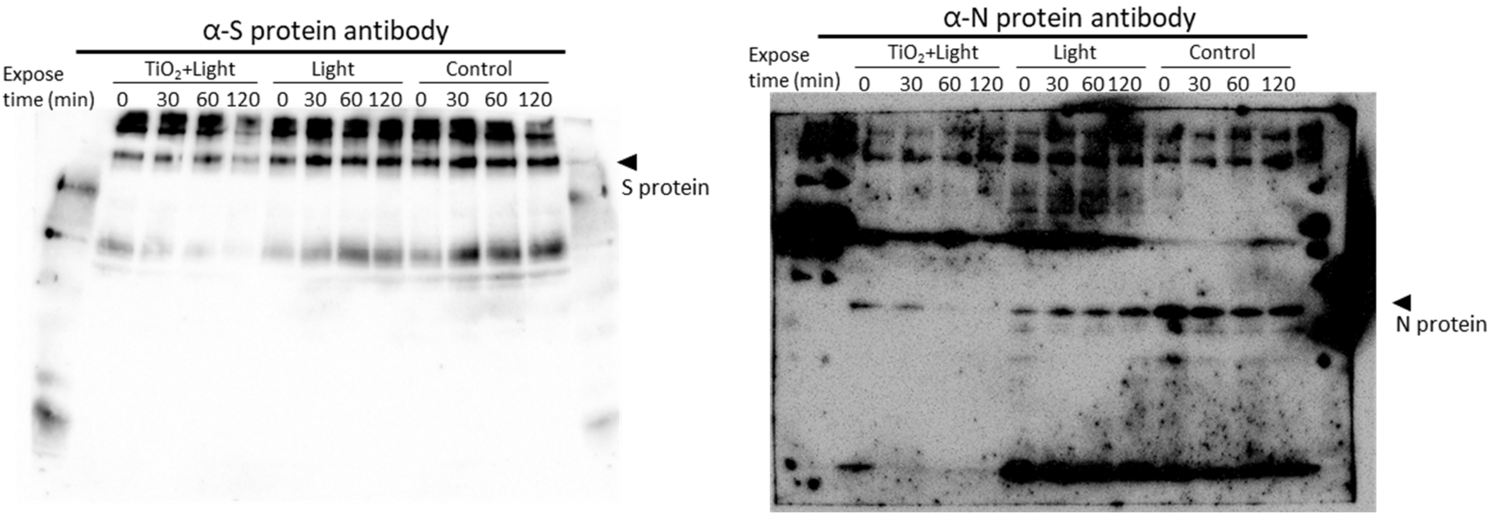

Figure S1. Original Images for Blots. Immunoblot images of viral spike (S) protein and nucleocapsid (N) proteins of SARS-CoV-2 treated with photocatalytic reaction. SARS-CoV-2 virus 1 mL with titer 1.78×10^6 $TCID_{50}$/mL on TiO_2-coated sheet were treated with photocatalytic reaction for 0, 30, 60 and 120 minutes. Positions of S and N proteins are indicated.
